# Mucosal-Associated Invariant T Cell Is a Potential Marker to Distinguish Fibromyalgia Syndrome from Arthritis

**DOI:** 10.1371/journal.pone.0121124

**Published:** 2015-04-08

**Authors:** Chie Sugimoto, Takahiko Konno, Rika Wakao, Hiroko Fujita, Hiroyoshi Fujita, Hiroshi Wakao

**Affiliations:** 1 Department of Hygiene & Cellular Preventive Medicine, Graduate School of Medicine, Hokkaido University, Sapporo, 060–8638, Japan; 2 Department of Rheumatology, Tokeidai-Memorial Clinic, Sapporo, 060–0031, Japan; 3 Pharmaceutical and Medical Device Agency (PMDA), Kasumigaseki, Tokyo, 100–0013, Japan; Karolinska Institutet, SWEDEN

## Abstract

**Background:**

Fibromyalgia (FM) is defined as a widely distributed pain. While many rheumatologists and pain physicians have considered it to be a pain disorder, psychiatry, psychology, and general medicine have deemed it to be a syndrome (FMS) or psychosomatic disorder. The lack of concrete structural and/or pathological evidence has made patients suffer prejudice that FMS is a medically unexplained symptom, implying inauthenticity. Furthermore, FMS often exhibits comorbidity with rheumatoid arthritis (RA) or spondyloarthritis (SpA), both of which show similar indications. In this study, disease specific biomarkers were sought in blood samples from patients to facilitate objective diagnoses of FMS, and distinguish it from RA and SpA.

**Methods:**

Peripheral blood mononuclear cells (PBMCs) from patients and healthy donors (HD) were subjected to multicolor flow cytometric analysis. The percentage of mucosal-associated invariant T (MAIT) cells in PBMCs and the mean fluorescent intensity (MFI) of cell surface antigen expression in MAIT cells were analyzed.

**Results:**

There was a decrease in the MAIT cell population in FMS, RA, and SpA compared with HD. Among the cell surface antigens in MAIT cells, three chemokine receptors, CCR4, CCR7, and CXCR1, a natural killer (NK) receptor, NKp80, a signaling lymphocyte associated molecule (SLAM) family, CD150, a degrunulation marker, CD107a, and a coreceptor, CD8β emerged as potential biomarkers for FMS to distinguish from HD. Additionally, a memory marker, CD44 and an inflammatory chemokine receptor, CXCR1 appeared possible markers for RA, while a homeostatic chemokine receptor, CXCR4 deserved for SpA to differentiate from FMS. Furthermore, the drug treatment interruption resulted in alternation of the expression of CCR4, CCR5, CXCR4, CD27, CD28, inducible costimulatory molecule (ICOS), CD127 (IL-7 receptor α), CD94, NKp80, an activation marker, CD69, an integrin family member, CD49d, and a dipeptidase, CD26, in FMS.

**Conclusions:**

Combined with the currently available diagnostic procedures and criteria, analysis of MAIT cells offers a more objective standard for the diagnosis of FMS, RA, and SpA, which exhibit multifaceted and confusingly similar clinical manifestations.

## Introduction

Fibromyalgia (FM) is defined as a widely distributed pain. FM is generally considered to be a pain disorder in the rheumatologists and the pain clinicians. In other clinical fields such as psychiatry, psychology, psychosomatic and probably general medicine, however, FM is regarded to be a syndrome (FMS) or psychosomatic disorder [1,2]. Although patients suffer an authentic widespread pain, physicians often consider FMS as somatoform disorders [3]. The concept that FMS is medically unexplained symptom, and the fact that FMS manifests symptoms unaccounted for by pathological findings devoid of biochemical and/or cellular markers have rendered the patient 's quality of life mediocre due to the failure of sound diagnosis and of appropriate treatments. The etiology and the pathology of FMS have remained enigmatic until present, though it has been proposed that FMS consists of central sensitivity syndromes that manifest as pain hypersensitivity and probably involves inflammation [4–8]. Diagnosis of FMS is further complicated by the fact that rheumatoid arthritis (RA) and spondyloarthritis (SpA) also exhibit a similar clinical manifestation; a great pain. This often causes misdiagnoses due, in part, to the empirical and/or subjective procedures in FMS diagnosis, based on the 2010 American College of Rheumatology (ACR) criteria and to the absence of the concrete biomarkers for FMS. The ACR criteria take into account a widespread pain index and the severity of the symptoms characteristic of FMS such as fatigue, non-refreshing sleep, cognitive problems, and the extent of somatic symptom reporting [9]. However, the absence of objective parameters in the diagnosis for FMS has made it difficult to distinguish it from other inflammation-related diseases, such as RA and SpA set forth. RA is characterized by synovial inflammation accompanied by progressive joint damage with chronic pain, systemic inflammation, and autoantibodies [10]. Structural variations, such as single-nucleotide polymorphisms in more than 30 genetic regions, are known in RA, bringing the risk of disease development to ~50%, while smoking also increases the incidence. SpA is a group of related but phenotypically distinct afflictions, including psoriatic arthritis, reactive arthritis, arthritis related to inflammatory bowel disease (IBD), a subtype of juvenile idiopathic arthritis, and ankylosing arthritis. It covers many clinical features including spinal aspects, peripheral arthritis, enthesopathy, and extra-articular features, such as uveitis, psoriasis, and IBD [11]. Axial SpA is diagnosed by the presence of sacroiliitis on imaging, plus several features of SpA or harboring human leukocyte antigen (HLA)-B27 with more than two features of SpA. However, early diagnoses are far from common in clinical practice because clear evidence of sacroiliitis is often not readily obtainable for the first 6–8 years and only a small subset of the population with HLA-B27 develops ankylosing arthritis [11,12]. Given the prevalence of FMS in patients with somatic damage ranges from 12 to 20% in RA and from 11 to 50% in SpA, sound diagnosis and appropriate treatments are difficult at an early stage [7]. Consequently, exploiting novel biomarkers for FMS to differentiate it from RA and SpA is invaluable.

Mucosal-associated invariant T (MAIT) cells are a subset of innate-type T lymphocytes, bridging innate and adaptive immunity and are mostly CD8^+^ or double negative (DN) harboring neither CD4 nor CD8, with few CD4^+^ cells [13]. Recent studies have revealed that bacteria-born vitamin B2 metabolites are antigens for MAIT cells [14]. While MAIT cells play a pivotal role in host defenses against bacterial and fungal infection, recent reports have suggested that MAIT cells also play roles in autoimmune diseases, such as IBD, multiple sclerosis, collagen-induced arthritis, and systematic lupus erythematosus (SLE), whose common hallmark is inflammation [15–18]. Given that MAIT cells are extremely abundant in human blood, intestinal lamina propria lymphocytes, and liver where they represent 1–8%, 1–10%, and 20–45% of all T lymphocytes, respectively, also produce an array of inflammatory cytokines and chemokines, and are implicated in the inflammatory diseases as described above [13,19], it seemed plausible that they could serve as a marker in autoimmune or autoinflammatory disease, such as RA, SpA, and probably FMS.

## Methods

### Patients

The subjects consisted of 26 FMS, 21 RA, and 37 SpA patients, and 16 HD. Characteristics of the patients are summarized in Table 1. All FMS patients met the ACR criteria [9,10]. Patients having comorbidity, such as HIV, diabetes, peripheral neuropathy, demyelinating disorders including multiple sclerosis, and inflammatory rheumatic diseases, such as RA, SpA, and polymyalgia rheumatica, were excluded from the FMS group. RA was diagnosed according to the 1987 ACR criteria [10]. All SpA patients satisfied the standard set by the European Spondylarthropathy Study Group and/or those modified by the New York criteria [11]. All RA, SpA, and FMS patients received no biological treatments (e.g., anti-TNF-α or anti-IL-6 monoclonal antibodies). No SpA patient carried HLA-B 27.

**Table 1:** Characteristics of the patients.

	FMS	RA	SpA	HD
Number of the patients	26	21	37	16
SpA subgroups, N				
AS^1^	N/A^8^	N/A	17	N/A
uSpA^2^	N/A	N/A	17	N/A
PAO^3^	N/A	N/A	1	N/A
Re-Arth^4^	N/A	N/A	2	N/A
Age at sampling, mean ± SD (years)	46.4 ± 14.0	59.8 ± 12.8	52.5 ± 11.3	45.0 ± 12.4
Sex, N male/female	1/25	2/19	6/31	2/14
FIQ^5^ score, median (25th-75th percentile)	30.6 ± 28.2	N/A	N/A	N/A
BASDAI^6^, median (25th-75th percentile)	N/A	N/A	5.2 ± 2.0	N/A
BASFI^7^, median (25th-75th percentile)	N/A	N/A	4.1 ± 2.4	N/A
Ongoing treatments, (% of the patients)				
methotrexate	0.0	71.4	16.2	N/A
sulfasalazine	0.0	9.5	51.4	N/A
cortico steroid	11.5	57.1	48.6	N/A
anti-convulsant	61.5	0.0	24.3	N/A
anti-depressant	42.3	4.8	5.4	N/A
opiate	38.5	0.0	29.7	N/A
neurotropin	61.5	19.0	10.8	N/A
others	0.0	23.8	0.0	N/A
no treatment	3.8	0.0	0.0	N/A

SpA was subtyped as indicated. Disease indexes, such as FIQ, BASDAI, and BASFI, are shown where applicable. HD: healthy donors, FMS: fibromyalgia syndrome, RA: rheumatoid arthritis, SpA: spondyloarthritis. Data are shown with median (25th-75th percentile), except the age data with mean (± standard deviation (SD)). Almost all FMS patients were on some treatment.

^1^AS, ankylosing arthritis

^2^uSpA, undifferentiated spondyloarthritis

^3^PAO, pustulotic arthro-osteitis

^4^Re-Arth, reactive arthritis

^5^FIQ, fibromyalgia Impact Questionnaire

^6^BASDAI, bath ankylosing spondylitis disease activity index

^7^BASFI, bath ankylosing spondylitis functional index

^8^N/A, not applicable

### Ethics approval

Institutional review board or ethics committee approval (Graduate School of Medicine, Hokkaido University and Tokeidai Memorial Clinic) and patient written informed consent were obtained before study participation according to the Declaration of Helsinki.

### Samples

Peripheral blood mononuclear cells (PBMCs) from FMS, RA, SpA, and HD were prepared using a Ficoll gradient and subjected to 8-color fluorescence-activated cell sorting (FACS) analysis, as described previously except that the MACSQuant (Miltenyi, Germany) equipped with a 605 nm filter was used [19]. Cell surface antigen expression was analyzed with the indicated phycoerythrin (PE)-labeled anti-human antibody within Brilliant Violet 421-labeled CD3^+^, Allophycocyanin (APC)-labeled CD161^+^, and PE/Cy7- or fluorescein isothiocyanate-labeled (FITC) anti-Vα7.2 (3C10)^+^-subset. The reaction mixture also contained Brilliant Violet 605-labeled CD4, APC/Cy7-labeled CD8, and FITC- or PE/Cy7-labeled CD45RO. A complete list of PE-labeled cell surface antigens used is provided in S1 Table. Drug treatment was interrupted for 48 h prior to the sample preparation for 9 FMS patients.

### Statistics

Statistical analyses of FACS data were performed with GraphPad Prism (ver. 6), and the significance of differences in expression on the cell surface antigen was evaluated with the nonparametric Mann-Whitney U test, the Kruskal-Wallis test, and the Wilcoxon matched-pairs signed rank test. *P* values were adjusted with the Dunn's multicomponent test where required. *P* values < 0.05 were considered to indicate statistical significance.

## Results and Discussion

**Fig 1:**
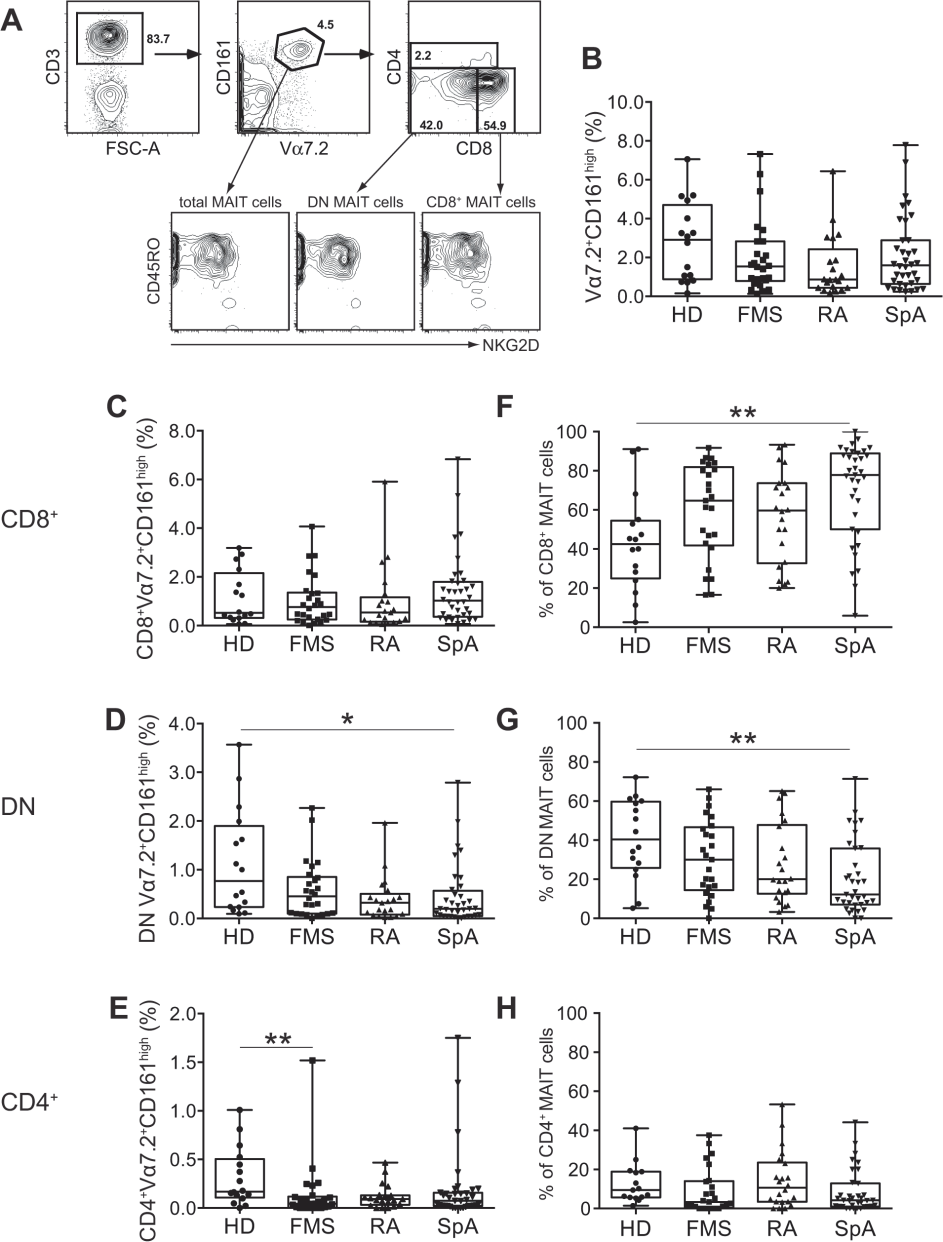
MAIT cells in HD, FMS, RA, and SpA. A, Representative FACS profile of MAIT cells and NKG2D expression in total, CD8^+^, and DN MAIT cells in PBMC from a FMS patient. The number in the figure shows the percentage of the populations. MAIT cells are defined as Vα7.2^+^CD161^high^ within CD3^+^ cells. B, The frequency of total MAIT cells in HD (n = 16), FMS (n = 26), RA (n = 21), and SpA (n = 36, missing one sample). The percentage of MAIT cells (Vα7.2^+^CD161^high^) within total T cells (CD3^+^) is shown. C, The frequency of CD8^+^ MAIT cells in HD, FMS, RA, and SpA. The percentage of CD8^+^ MAIT cells (Vα7.2^+^CD161^high^CD8^+^) within total T cells (CD3^+^) is shown. D, The frequency of DN MAIT cells in HD, FMS, RA, and SpA. The percentage of DN MAIT cells (Vα7.2^+^CD161^high^DN) within total T cells (CD3^+^) is shown. E, The frequency of CD4^+^ MAIT cells in HD, FMS, RA, and SpA. The percentage of CD4^+^MAIT cells (Vα7.2^+^CD161^high^CD4^+^) within total T cells (CD3^+^) is shown. F, The percentage of CD8^+^ MAIT cells (CD8^+^Vα7.2^+^CD161^high^) among total MAIT cells (Vα7.2^+^CD161^high^) in HD, FMS, RA, and SpA. G, The percentage of DN MAIT cells (CD8^-^CD4^-^Vα7.2^+^CD161^high^) among total MAIT cells (Vα7.2^+^CD161^high^) in HD, FMS, RA, and SpA. H, The percentage of CD4^+^ MAIT cells (CD4^+^Vα7.2^+^CD161^high^) among total MAIT cells (Vα7.2^+^CD161^high^) in HD, FMS, RA, and SpA. B-H. All data are presented as median. Horizontal line: Median; boxes: 25th percentile and 75th percentile; whiskers: Minimum and Maximum. Asterisk shows the group-pair exhibiting significance. *: *P*< 0.05, **: *P* < 0.01 (*P* value adjusted with the Dunn's multicomponent test after the Kruskal-Wallis test).

Given that FMS features widespread pain, fatigue, and distressed mood, it has been believed that inflammatory cytokines play a role in triggering neuroendocrine aberrations, eventually leading to these symptoms. Accordingly, some reports have demonstrated the aberrant expression of inflammatory cytokines in FMS [4,20,21]. Nevertheless, the source of cytokines remained unidentified, counting against their potential utility as a biomarker. Thus, we analyzed MAIT cells that produce a plethora of the inflammatory cytokines and chemokines [19]. A representative FACS profile of MAIT cells and concomitant cell surface antigen expression (NKG2D, a NK receptor) from a FMS patient is shown in Fig. 1A. We then compared the percentage of total MAIT cells (defined as Vα7.2^+^CD161^high^ cells within CD3^+^ cells) in the diseases, and found that they represented [median (25th percentile; 75th percentile): 2.9% (0.9; 4.7), 1.5% (0.8; 2.8), 0.9% (0.4; 2.4), 1.6% (0.6; 2.9] in HD, FMS, RA, and SpA, respectively (Fig. 1B and S2A Table). However, there was no statistical significance in difference of the MAIT cell populations among diseases. Because MAIT cells consist of primarily CD8^+^ and double negative (DN), and few CD4^+^ cells [13], each subset was further analyzed. Difference in MAIT cell frequency was seen in CD4^+^ and DN MAIT cells after the Kruskal-Wallis test (S2B Table). *P* value adjustment uncovered that there was a difference in frequency of DN MAIT cells between HD and SpA and that of CD4^+^ MAIT cells between HD and FMS (Fig. 1D-E and S2A–B Table). When the proportion of CD8^+^, DN, and CD4^+^ MAIT cells were analyzed within Vα7.2^+^CD161^high^ cells (considered to be total MAIT cells) in different settings, a significant increase in CD8^+^ MAIT cells concomitant with a decrease in DN MAIT cells was observed in SpA as compared with HD (Fig. 1F-G). This suggested that SpA is characterized by the increase in proportion of CD8^+^MAIT cells that is most likely counterbalanced by the decrease of DN MAIT cells. We then focused our study on total, CD8^+^, and DN MAIT cells for cell surface antigen analysis, as CD4^+^ MAIT cells were rare.

**Fig 2:**
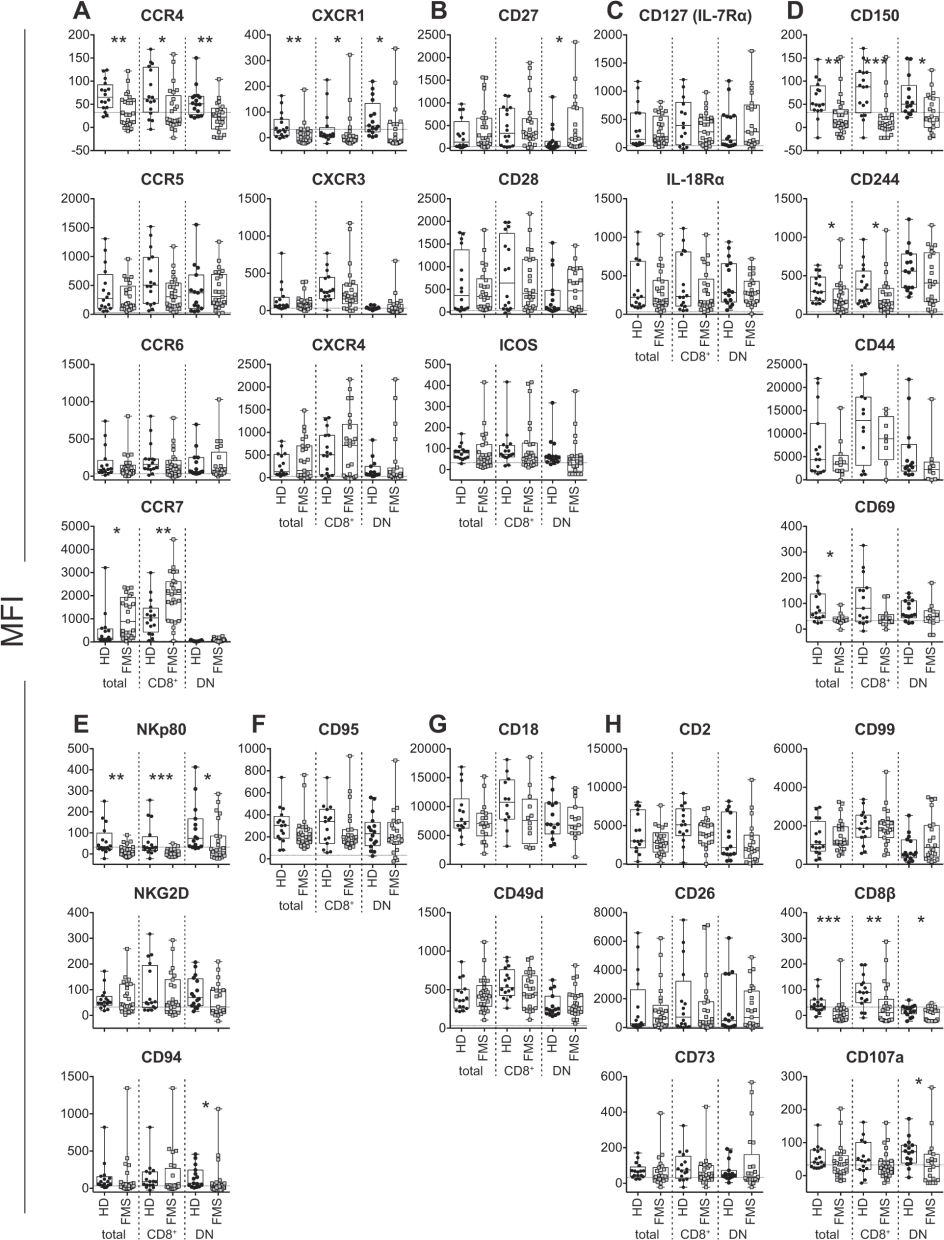
Comparison of the cell surface antigen expression level in MAIT cells between HD and FMS. A, Chemokine receptor expression in total, CD8^+^, and DN MAIT cells. B, Co-stimulatory molecule expression in total, CD8^+^, and DN MAIT cells. C, Cytokine receptor expression in total, CD8^+^, and DN MAIT cells. D, SLAM family, memory, and activation marker expression in total, CD8^+^, and DN MAIT cells. E, NK receptor expression in total, CD8^+^, and DN MAIT cells. F, CD95 (Fas) expression in total, CD8^+^, and DN MAIT cells. G, Integrin family expression in total, CD8^+^, and DN MAIT cells. H, Miscellaneous molecule expression in total, CD8^+^, and DN MAIT cells. A-H. MFI is shown with median. The dotted line indicates MFI for the isotype control. Horizontal line: Median; boxes: 25th percentile and 75th percentile; whiskers: Minimum and Maximum. Asterisk shows the group-pair exhibiting significance. *: *P*< 0.05, **: *P* < 0.01, ***: *P*<0.001 (the nonparametric Mann-Whitney U test)

We sought the cell surface antigens in MAIT cells that allowed the distinction between HD and FMS. In FMS, we found a significant increase of CCR7, a chemokine receptor required for lymph node homing, in total MAIT cells and in CD8^+^ MAIT cells and of CD27, a costimulatory molecule for T cell activation, in DN MAIT cells, compared with HD (Fig. 2 and Table 2 and S3 Table). In contrast, there was a decrease in two chemokine receptors, CCR4, CXCR1, a natural killer (NK) receptor, NKp80, a signaling lymphocyte associated molecule (SLAM) family, CD150, and a coreceptor, CD8β in all subsets of MAIT cells, while a decrease in CD244, another SLAM family member, in total and CD8^+^ MAIT cells, CD69, an activation marker, in total MAIT cells, and CD107a in DN MAIT cells, was seen compared with HD (Fig. 2 and Table 2 and S3 Table).

**Table 2:** Cell surface antigens showing a difference in expression in MAIT cells between HD and FMS.

categories	antigens	Mann-Whitney (HD vs. FMS)		
		MAIT cells		
		total	CD8^+^	DN
chemokine receptors	CCR4	0.0033**	0.0282*	0.0021**
	CCR5	ns	ns	ns
	CCR6	ns	ns	ns
	CCR7	0.0103*	0.0062**	ns
	CXCR1	0.0024**	0.0207*	0.0148*
	CXCR3	ns	ns	ns
	CXCR4	ns	ns	ns
costimulators	CD27	ns	ns	0.0412*
	CD28	ns	ns	ns
	ICOS	ns	ns	ns
cytokine receptors	CD127 (IL-7Rα)	ns	ns	ns
	IL-18Rα	ns	ns	ns
NK receptors	CD94	ns	ns	ns
	NKp80	0.0034**	0.0004***	0.0153*
	NKG2D	ns	ns	ns
SLAM family	CD150	0.0014**	0.0001***	0.0459*
	CD244	0.0250*	0.0354*	ns
activation	CD69	0.0190*	ns	ns
memory	CD44	ns	ns	ns
Fas	CD95	ns	ns	ns
integrin family	CD18	ns	ns	ns
	CD49d	ns	ns	ns
relevant to MAIT cells	CD26	ns	ns	ns
	CD8β	0.0004***	0.0062**	0.0171*
immuno-regulatory molecule	CD73	ns	ns	ns
miscellaneous	CD2	ns	ns	ns
	CD99	ns	ns	ns
degranulation	CD107a	ns	ns	0.0120*

*P* values after comparison between FMS and HD (nonparametric Mann-Whitney U-test) are shown. The category of the cell surface antigens is indicated. ns: not significant. Total: total MAIT cells, CD8^+^: CD8^+^ MAIT cells, DN: DN MAIT cells.

**Fig 3:**
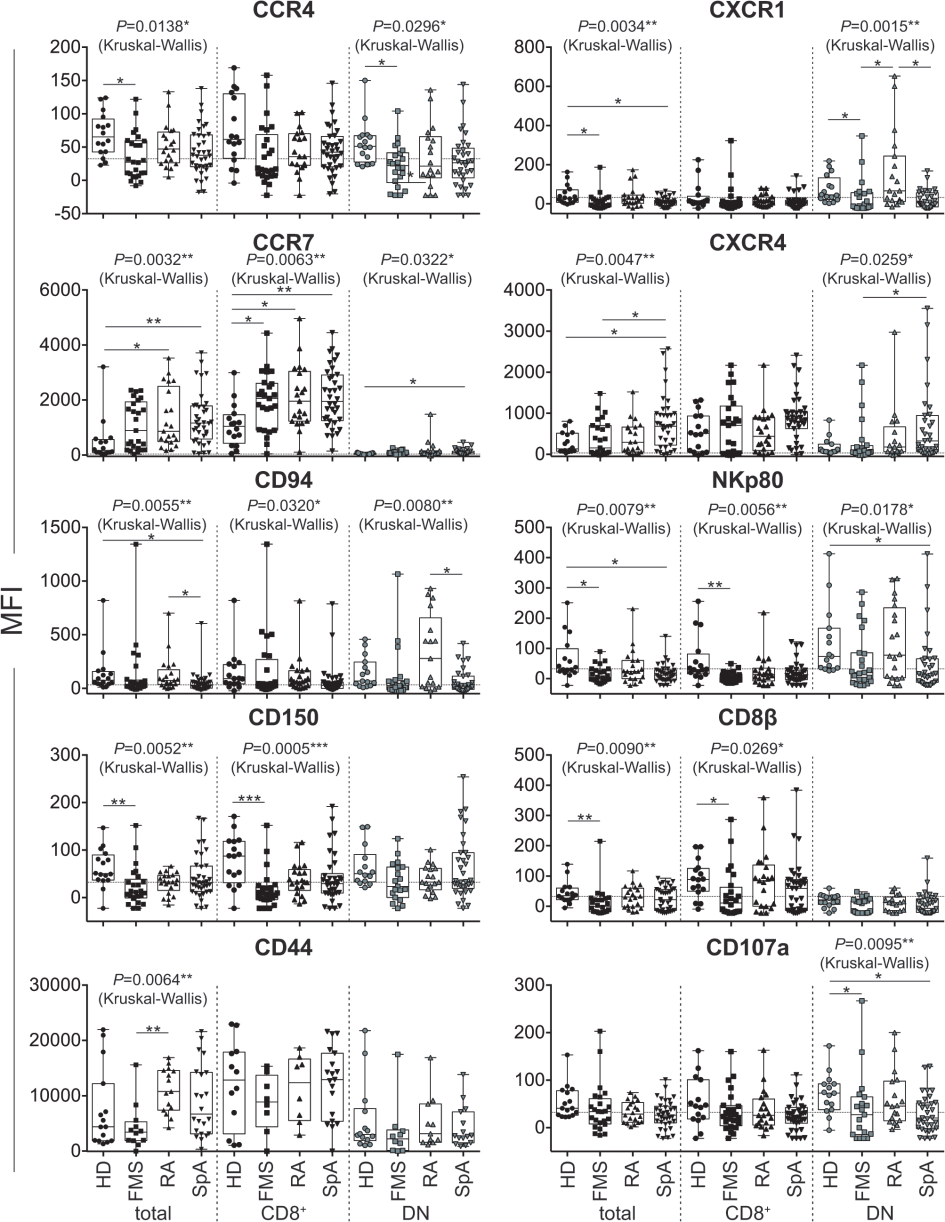
Potential biomarkers distinguishing HD, FMS, RA and SpA. MFI is shown with median for the indicated cell surface antigen. The dotted line indicates MFI for the isotype control. Horizontal line: Median; boxes: 25th percentile and 75th percentile; whiskers: Minimum and Maximum. The number in figure shows a *P* value after the Kruskal-Wallis test. Asterisk shows the group-pairs exhibiting significance. *: *P*< 0.05, **: *P* < 0.01, ***:*P*<0.001 (*P* value adjusted with the Dunn's multicomponent test). total: total MAIT cells; CD8^+^; CD8^+^ MAIT cells; DN: DN MAIT cells.

Next we tried to find out the cell surface antigens in MAIT cells that could differentiate HD, FMS, RA, and SpA. Kruskal-Wallis test has revealed that CCR4, CCR7, CXCR1, CXCR4, CD94, NKp80, CD150, CD44, CD8β, and CD107a are possible makers to distinguish the three diseases (Table 3, Kruskal-Wallis test). Multiple comparisons after *P* value adjustment have allowed the identification of CCR4, CCR7, CXCR1, NKp80, CD150, CD8β and CD107a to be potential primary markers for FMS to distinguish from HD, RA and SpA (Fig. 3 and Table 3, Adjusted *P* values). In addition, CXCR1 in DN MAIT cells and CD44 in total MAIT cells may serve as auxiliary markers to differentiate FMS from RA (Fig. 3 and Table 3 and S3 Table). CXCR4 appeared to be a marker to distinguish SpA from HD in total MAIT cells, and also be useful to discern FMS and SpA in total and DN MAIT cells (Fig. 3 and Table 3 and S3 Table). Among the cell surface molecules so far studied, CD94 in total and DN MAIT cells, and CXCR1 in DN MAIT cells, would allow the distinction between RA and SpA (Fig. 3 and Table 3 and S3 Table).

**Table 3:** Cell surface antigens distinguishing HD, FMS, RA, and SpA.

categories	antigens	Kruskal-Wallis (HD, FMS, RA, and SpA)			Adjusted P value					
		MAIT cells			MAIT cells					
		total	CD8^+^	DN	total		CD8^+^		DN	
chemokine receptors	CCR4	0.0138*	ns	0.0296*	HD vs. FMS	0.0111*	ns		HD vs. FMS	0.0269*
	CCR5	ns	ns	ns	ns		ns		ns	
	CCR6	ns	ns	ns	ns		ns		ns	
	CCR7	0.0032**	0.0063**	0.0322*	HD vs. FMS	0.0102*	HD vs. FMS	0.0347*	HD vs. SpA	0.0333*
					HD vs. RA	0.0026**	HD vs. RA	0.0186*		
							HD vs. SpA	0.0062**		
	CXCR1	0.0034**	ns	0.0015**	HD vs. FMS	0.0140*	ns		HD vs. FMS	0.0474*
					HD vs. SpA	0.0143*			FMS vs. RA	0.0169*
									RA vs. SpA	0.0237*
	CXCR3	ns	ns	ns	ns		ns		ns	
	CXCR4	0.0047**	ns	0.0259*	HD vs. SpA	0.0438*	ns		FMS vs. SpA	0.0201*
					FMS vs. SpA	0.0161*				
costimulators	CD27	ns	ns	ns	ns		ns		ns	
	CD28	ns	ns	ns	ns		ns		ns	
	ICOS	ns	ns	ns	ns		ns		ns	
cytokine receptors	CD127 (IL-7Rα)	ns	ns	ns	ns		ns		ns	
	IL-18Rα	ns	ns	ns	ns		ns		ns	
NK receptors	CD94	0.0055**	0.0320*	0.0080**	HD vs. SpA	0.0191*	ns		RA vs. SpA	0.0304*
					RA vs. SpA	0.0365*				
	NKp80	0.0079**	0.0056**	0.0178*	HD vs. FMS	0.0138*	HD vs. FMS	0.0025**	HD vs. SpA	0.0362*
					HD vs. SpA	0.0189*				
	NKG2D	ns	ns	ns	ns		ns		ns	
SLAM family	CD150	0.0052**	0.0005***	ns	HD vs. FMS	0.0022**	HD vs. FMS	0.0002***	ns	
	CD244	ns	ns	ns	ns		ns		ns	
activation	CD69	ns	ns	ns	ns		ns		ns	
memory	CD44	0.0064**	ns	ns	FMS vs. RA	0.0052**	ns		ns	
Fas	CD95	ns	ns	ns	ns		ns		ns	
integrin family	CD18	ns	ns	ns	ns		ns		ns	
	CD49d	ns	ns	ns	ns		ns		ns	
relevant to MAIT cells	CD26	ns	ns	ns	ns		ns		ns	
	CD8β	0.0090**	0.0269*	ns	HD vs. FMS	0.0095**	HD vs. FMS	0.0336*	ns	
immuno-regulatory molecule	CD73	ns	ns	ns	ns		ns		ns	
miscellaneous	CD2	ns	ns	ns	ns		ns		ns	
	CD99	ns	ns	ns	ns		ns		ns	
degranulation	CD107a	ns	ns	0.0095**	ns		ns		HD vs. FMS	0.0390*
									HD vs. SpA	0.0205*

*P* values for the indicated antigens as a diagnostic indicator after all possible multiple comparisons between the groups (Kruskal-Wallis test), and after adjustment with the Dunn's multicomponent test (Adjusted *P* value) are shown. Group pairs showing a significant difference are also depicted (Adjusted *P* value). ns: not significant. total: total MAIT cells, CD8^+^: CD8^+^ MAIT cells, DN: DN MAIT cells.

**Fig 4:**
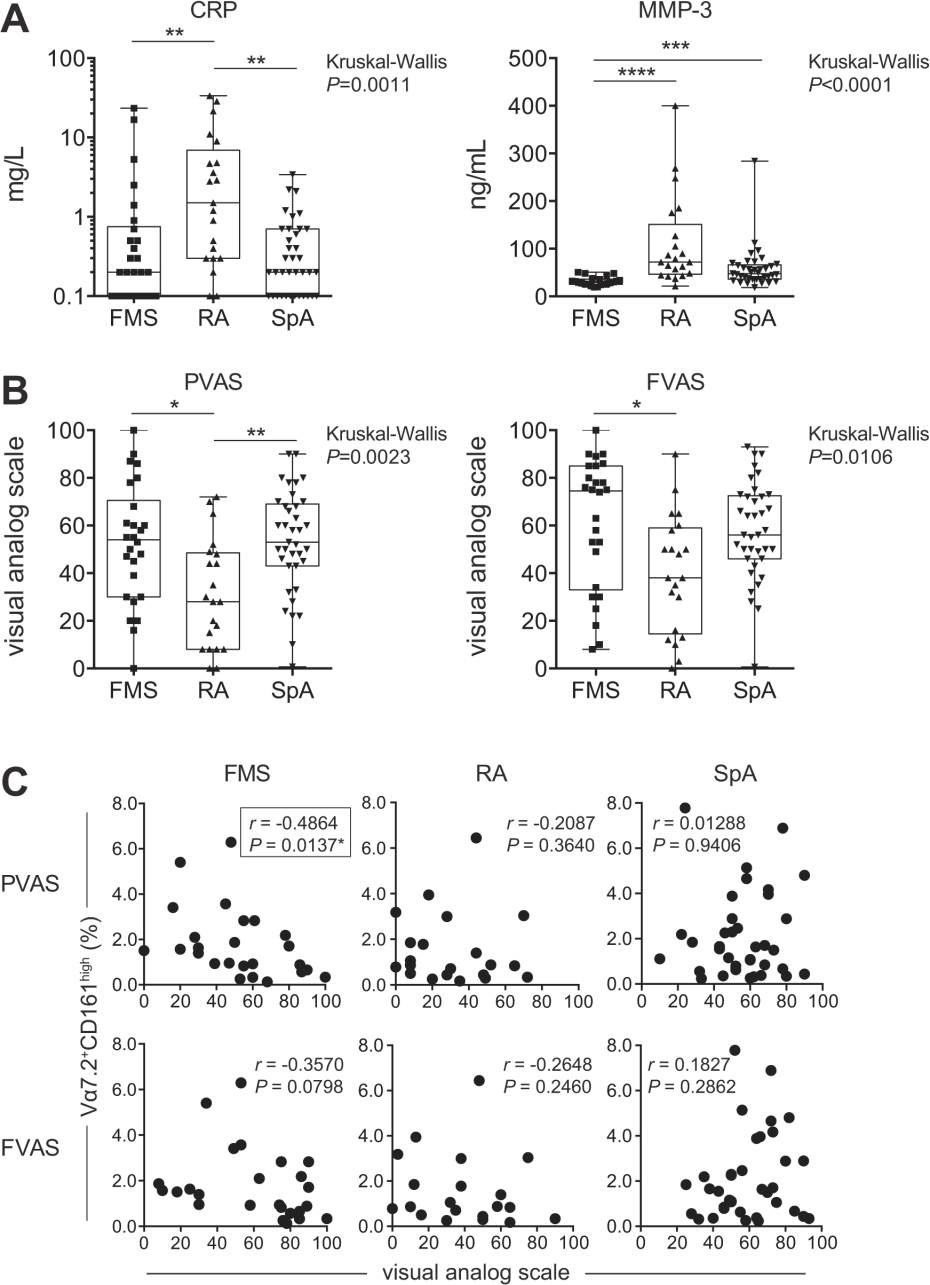
Biochemical and physical parameters in FMS, RA, and SpA. A: Serum CRP concentrations in FMS, RA, and SpA (left panel).: Serum MMP-3 concentrations in FMS, RA, and SpA (right panel). B: PVAS in FMS, RA, and SpA (left panel).: FVAS in FMS, RA, and SpA (right panel). A-B. Horizontal line: Median; boxes: 25th percentile and 75th percentile; whiskers: Minimum and Maximum. Asterisk shows the group-pairs exhibiting significance. *: *P*< 0.05, **: *P* < 0.01, ***: *P*<0.001 (*P* value adjusted after the Kruskal-Wallis test with the Dunn's multicomponent test). C: Correlation between PVAS/FVAS and MAIT cell percentage (% of Vα7.2^+^CD161^high^ cells among CD3^+^ cells) in a cohort of 26 FMS (left panels), of 21 RA (middle panels), and of 36 SpA (right panels) patients. The correlation was analyzed with the Spearman rank correlation test. *: *P*< 0.05, r: correlation coefficient.

Analysis of serum showed that RA had elevated levels of C-reactive protein (CRP) compared with FMS and/or SpA (Fig. 4A and S4A–B Table). Matrix metalloproteinase (MMP)-3 level in FMS was significantly lower than that in RA and SpA (Fig. 4A and S4A–B Table). Thus, CRP and MMP-3 might be potential biomarkers for distinguishing the diseases. Despite biomarkers such as CRP and rheumatoid factors, 20–50% of RA patients are devoid of them [10]. This means that there is a continuing need for a novel biomarker(s) for RA. In this respect, CCR7 may be a potential one for RA to distinguish from HD in total MAIT cells, and in CD8^+^ MAIT cells (Fig. 3 and Table 3 and S3 Table). When pain visual analog scale (PVAS) was compared, FMS and SpA showed a greater value than RA (Fig. 4B and S4A–B Table). Similarly, FMS exhibited a greater fatigue visual analog scale (FVAS) than RA (Fig. 4B and S4A–B Table). Intriguingly, there existed an inversed correlation between PVAS and the MAIT cell frequency in FMS, suggesting that MAIT cells would somehow be implicated in the pathology of FMS (Fig. 4C). Combined with data from the cell surface antigen analysis in MAIT cells, CRP, MMP-3, PVAS and FVAS may serve as auxiliary markers to distinguish FMS from RA and/or SpA.

**Fig 5:**
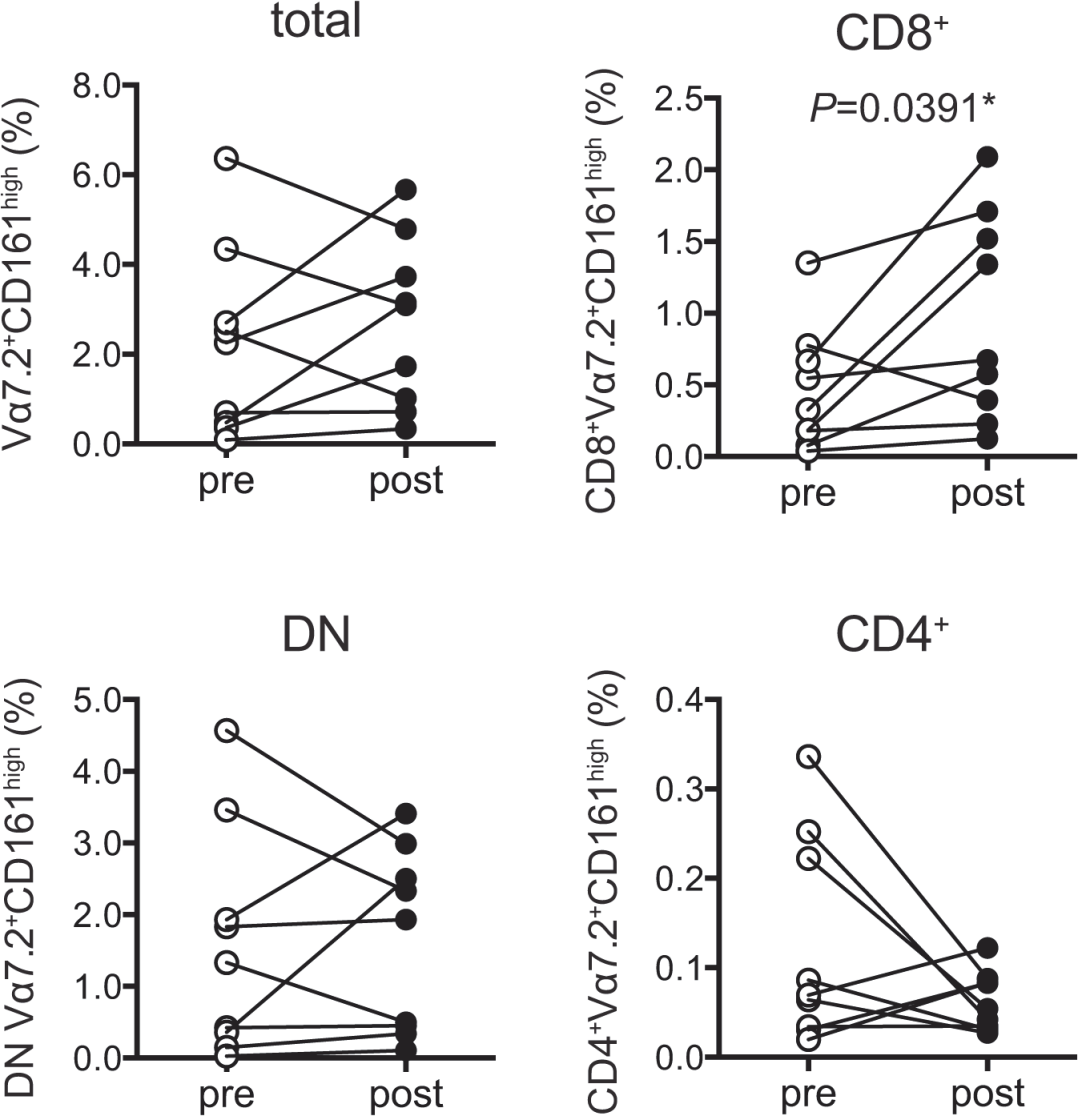
Effect of the daily drug treatment on MAIT cell frequency in FMS. The percentage of total, CD8^+^, DN, and CD4^+^ MAIT cells (Vα7.2^+^CD161^high^) within total T cells (CD3^+^) from the same individuals (n = 9) before and after the drug treatment interruption is shown. The statistical significance and *P* value were with the Wilcoxon matched-pairs signed rank test. Asterisk shows significance. *: *P*< 0.05

**Fig 6:**
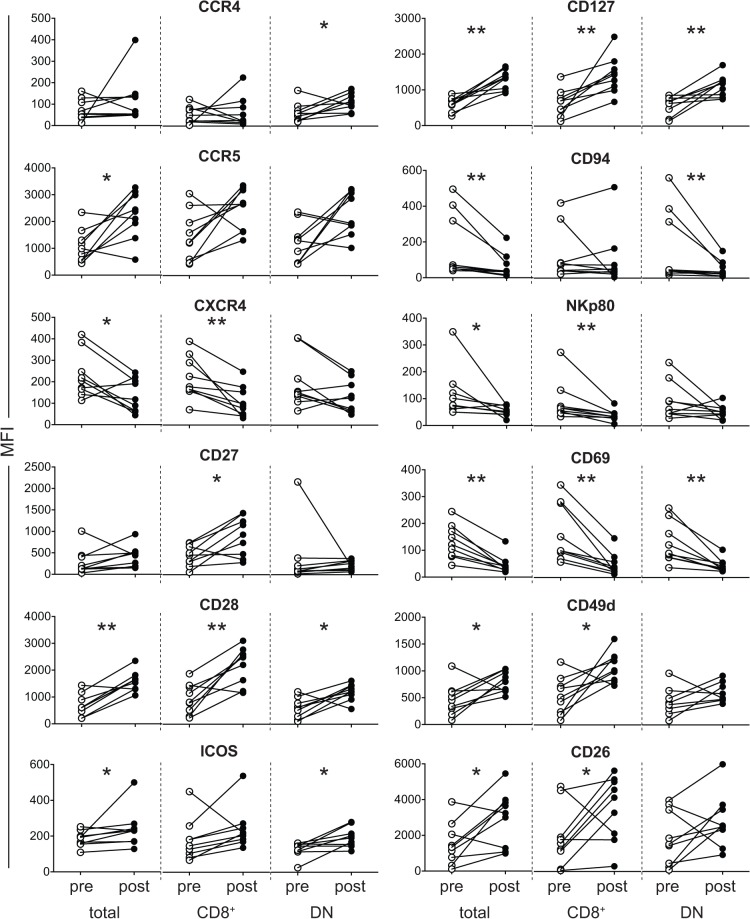
Effect of the daily drug treatment on MAIT cell surface antigens in FMS. MFI of the indicated antigen in total, CD8^+^, and DN MAIT cells from the same individual (n = 9) before and after the drug treatment interruption is shown. The statistical significance was assessed with the Wilcoxon matched-pairs signed rank test. Asterisk shows significance. *: *P*< 0.05, **: *P* < 0.01

We then assessed whether the daily drug intake affected the frequency of MAIT cell subsets and the expression of the cell surface molecules in MAIT cells from FMS, as patients were ongoing treatment when the above analysis was performed (Table 4). After 48 h of the drug treatment interruption, CD8^+^ MAIT cells have increased, while little change in CD4^+^, DN, and total MAIT cell frequency was observed, implying that CD8^+^ MAIT cells were particularly sensitive to the drugs and would tightly be linked with the morbidity of FMS (Fig. 5 and S5 Table). Such an interruption also engendered an increase of CCR4 in DN MAIT cells and of CCR5 in total MAIT cells, while CXCR4 expression has declined in total and CD8^+^ MAIT cells (Fig. 6 and S6 Table). Given that CCR4 and CCR5 are receptors for the inflammatory chemokines such as CCL3, CCL3L1, CCL4, CCL5, CCL7, CCL8, and CCL11, such an increase indicated that the morbidity of FMS comprises, in part, inflammation [20,21]. Interruption of the drug treatment enhanced CD27 level in CD8^+^ MAIT cells (Fig. 6 and S6 Table).

**Table 4:** Disease and medication history of the FMS patients analyzed in Figs. 5 and 6.

Patient ID	Disease duration (years)	Duration of medication (years)	pharmacological treatment				
			anti-depressant	anti-convulsant	opioid	steroid	NSAID
1	6.0	1.7	-	Pregabalin	Tramadol/Acetaminophen	-	-
2	5.0	3.6	Duloxetine	Pregabalin	Tramadol	-	-
3	3.0	2.1	-	Clonazepam	-	-	Naproxen
4	5.0	3.8	Duloxetine	Pregabalin Clonazepam	-	-	-
5	5.0	4.7	Duloxetine	Pregabalin	-	-	-
6	11.0	2.8	-	Clonazepam	-	-	-
7	9.0	1.7	-	Clonazepam	-	-	Aconite root powder
8	3.0	2.2	Duloxetine	-	-	-	-
9	4.0	1.9	Duloxetine	-	Tramadol	-	-

NSAID: Non-steroidal anti-inflammatory drugs

Likewise, CD28 expression increased in all subset of MAIT cells (Fig. 6 and S6 Table). Besides these, an increase of another costimulatory molecule ICOS was seen in total and DN MAIT cells (Fig. 6 and S6 Table). CD127 expression was also augmented in all MAIT cell subsets (Fig. 6 and S6 Table). These results suggested that MAIT cells in FMS possess an activated phenotype and that the elevated CD127 (IL-7 receptor alpha chain) expression would culminate in enhanced IL-17A and IFN-γ production [4,22]. CD94 and NKp80 showed an alternation in expression upon the drug treatment interruption. CD94 declined in total and DN MAIT cells, and also did NKp80 in total and CD8^+^ MAIT cells (Fig. 6 and S6 Table). As HLA-E polymorphism associates with ankylosing spondylitis [23], the decrease in CD94 expression would result in an attenuated CD94/NKG2A -HLA-E interaction, which in turn may be relevant to the etiology of FMS. The decrease of CD69 was also seen in all subset of MAIT cells (Fig. 6 and S6 Table). This indicates that the drug treatment interruption kept MAIT cells being resting state. In marked contrast, CD49d, an integrin family member and CD26, a dipeptidase have increased in total and CD8^+^ MAIT cells (Fig. 6 and S6 Table). Given that CD49d, the alpha 4 subunit of the very late antigen-4, has been shown to be responsible for chronic lymphocytic leukemia cell homing to the bone marrow and to the lymphoid organs and that MAIT cells are found in the inflammatory lesions within the brain from MS patients [24,25], it is predicted that MAIT cells altered the homing propensity through the molecule, and were actively implicated in the morbidity of FMS. The upregulation of CD26 contrasted sharply with what observed in CD4^+^ T cells in RA where methotrexate treatment had little effect on CD26 expression [25]. Since CD26 is responsible for truncation of many growth factors, chemokines, and cytokines, it is conceivable that such an increase of CD26 culminated in enhanced production of inflammatory mediators such as CCL11, IL-1, IL-6, and IL-8 in FMS [5,26–29]. Combined with the aberrant production of other inflammatory cytokines, these products would cause neuroendocrine anomaly, which eventually results in a widespread pain [4, 7, 21]. In this regard, it is tempting to speculate that MAIT cells which produce a plethora of inflammatory cytokines and chemokines are, at least in part, responsible for the pathology of FMS.

It is worthwhile to note that not all the antigens relevant to FMS have shown an alteration in expression upon drug treatment interruption. Indeed, the expression of CD150, CD244, CD8β, CCR7, and CD107a has not been affected (Tables 2 and 3, and data not shown). These data *in toto* corroborates the fact that several cell surface molecules in MAIT cells serves as a diagnostic marker, and further suggested that they are susceptible to a variety of drugs such as anti-convulsant, anti-depressant, opioid, and non-steroidal anti-inflammatory drugs (Table 4, and Figs. 5 and 6). Further study is warranted to decipher the molecular pathways and mechanisms underlying the link between the drug intake and the phenotypic change in MAIT cells. Such an analysis would shed light on the role of MAIT cells in the etiology and/or pathology of FMS that has been remained conundrum.

## Conclusion

MAIT cells served as a diagnostic marker for FMS, and could also be used to distinguish FMS from RA and SpA together with other biochemical and physical parameters. The dynamic change in expression of several cell surface molecules in MAIT cells before and after the drug treatment interruption most likely reflected the pathological states of FMS, which merits further study.

## Supporting Information

S1 Table List of the cell surface antigens (PE-labeled) used in the study. Asterisk indicates antigens for which results are shown in Figs. 2 and 3.
(XLSX)

S2 Table Statistics of the MAIT cell subset frequency. (A) The percentage of total, CD4^+^, CD8^+^, and DN MAIT cells among CD3^+^ T cells in HD, FMS, RA, and SpA. (B) *P* values after the Kruskal-Wallis test among the diseases and adjusted *P* values. *P* values for the difference in frequency of CD4^+^ and DN MAIT cells among the subjects are indicated after the Kruskal-Wallis test (middle column). The paired-groups exhibiting a difference in frequency of MAIT cell subsets are shown with *P* values adjusted with the Dunn's multicomponent test (Adjusted *P* value) (right column). Asterisk indicates significance. ns: not significant.
(XLSX)

S3 Table Statistics of the cell surface antigen MFI in total, CD8^+^, and DN MAIT cells from HD, FMS, RA, and SpA. Some samples are missing due to the rarity of MAIT cells. Cell surface antigens are categorized as shown. MFI for the indicated cell surface antigen is depicted. total MAIT cells, CD8^+^: CD8^+^ MAIT cells, DN: DN MAIT cells.
(XLSX)

S4 Table Statistics of PVAS, FVAS, CRP, and MMP-3 in FMS, RA and SpA. (A) Statistics of the physical indexes (PVAS and FVAS), and of the biochemical indexes (CRP and MMP-3). The number of subjects, and PVAS and FVAS score are shown. For CRP and MMP-3, serum concentration is indicated. (B) *P* values for PVAS, FVAS, CRP, and MMP-3. *P* values are calculated with the Kruskal-Wallis test. The paired-groups exhibiting a statistical difference are shown with *P* values adjusted with the Dunn's multicomponent test (Adjusted *P* value). Asterisk indicates significance.
(XLSX)

S5 Table Statistics of the MAIT cell subset frequency before and after the drug treatment interruption in FMS. The percentage of total, CD4^+^, CD8^+^, and DN MAIT cells (Vα7.2^+^CD161^high^) within the total T cells (CD3^+^) from the same individuals (n = 9) before (pre) and after (post) the drug treatment interruption is shown. *P* values are calculated with the Wilcoxon matched-pairs signed rank test. Asterisk indicates significance. *: *P*< 0.05 total: total MAIT cells, CD4^+^: CD4^+^ MAIT cells, CD8^+^: CD8^+^ MAIT cells, DN: DN MAIT cells.
(XLSX)

S6 Table Statistics of the cell surface antigen MFI in total, CD8^+^, and DN MAIT cells before (pre) and after (post) the drug treatment interruption in FMS. Cell surface antigens are categorized as shown. MFI of the indicated cell surface antigen from the same individuals (n = 9) is shown. total: total MAIT cells, CD8^+^: CD8^+^ MAIT cells, DN: DN MAIT cells. *P* values were calculated with the Wilcoxon matched-pairs signed rank test. Asterisk indicates significance. *: *P*< 0.05, **: *P* < 0.01
(XLSX)
